# Multi-beam array stitching method based on scanning Hartmann for imaging quality evaluation of large space telescopes

**DOI:** 10.1038/s41598-018-25632-0

**Published:** 2018-05-08

**Authors:** Haisong Wei, Haixiang Hu, Feng Yan, Xindong Chen, Qiang Cheng, Donglin Xue, Xuejun Zhang

**Affiliations:** 1 Key Laboratory of Optical System Advanced Manufacturing Technology, Changchun Institute of Optics, Fine Mechanics and Physics, Chinese Academy of Sciences, Changchun, Jilin 130033 China; 2 University of Chinese Academy of Sciences, Beijing, 100049 China

## Abstract

To test large-aperture space optical systems in a simple and highly efficient manner, the scanning Hartmann test (SHT) has been used to measure the sub-aperture wavefront slopes of optical systems by scanning with a collimated beam followed by retrieval of the overall wavefront form. However, the use of such a method contains a crucial flaw in that pointing errors of the translation stage can severely affect the test accuracy. Therefore, a multi-beam stitching method is proposed to correct pointing errors by stitching together data obtained by successive sub-aperture acquisition. In this paper, a test principle and a data processing method are detailed. Simulation results theoretically verify a high precision for the stitching algorithm. Furthermore, a multi-beam array stitching test system (MASTS) is developed and used to successfully test an actual space optical system of ∅800 mm. The MASTS shows a deviation of 1/50 λ (λ = 632.8 nm) root mean square (RMS) from the interferometric results and a repeatability of 1/80 λ RMS, which demonstrates high precision, high repeatability and low sensitivity to air turbulence compared to interferometric measurement. In future engineering applications, the MASTS has great potential to solve the test problems of space optical systems using ultra-large apertures.

## Introduction

The space-based optical telescope is one of the most significant astronomical instruments. For example, the well-known Hubble Space Telescope has served for 26 years and been used to record the lagest ever number of visible-light images, enabling a deep view into space and time^1,2^. The optical system is the core component of an astronomical telescope. The aperture sizes of space optical systems have been increased to achieve higher resolution and light collection capability.

Evaluation of the imaging quality of optical systems is a key link in the telescope manufacturing process. Test methods for such optical systems includes the star test, collimator test, and auto-collimating test. First, ground-based large aperture optical telescopes use starlight for alignment and wavefront correction. It determines the appropriate commands for control of the active optics system for optical alignment^3–5^. Second, the image quality of a finished space optical system is always evaluated using a large collimator before it is launched into space. After transport of a finished telescope from the laboratory to the launching site, its optical performance may change due to vibration. Thus, an outdoor test at the launching site is carried out as a final test procedure to ensure that the imaging quality of the telescope satisfies the needs of the project. The famous Hubble Space Telescope did not undergo an optical test before launch into space; thus, its flawed imaging quality remained undiscovered until after launch, causing tremendous losses. In the collimator test, a collimated beam provided by a large collimator is passed through the optical system and focused onto the focal plane. The detected spot is analyzed to evaluate the imaging quality of the optical system based on the modulation transfer function (MTF)^6^. As the aperture of the optical system increases, evaluation of the imaging quality by this method requires a collimator with a large aperture and a long focal length. Thus, the cost increases dramatically. For instance, the Large Optical Test and Integration Site (LOTIS) at the Lockheed Martin Space Systems Company (LMSSC) allows advanced optical tests for systems up to a maximum aperture of 6.5 meters. However, the construction of such a facility costs much money^7^. The third test method is the auto-collimating test, which involves the use of a plane mirror and an interferometer. However, the auto-collimating test cannot be used for a finished optical system, because the imaging sensor of optical system is mounted in the focal plane already and cannot be replaced with an interferometer. And it is very difficult to fabricate a plane mirror with an aperture size in excess of 3 meters. The Changchun Institute of Optics, Fine Mechanics and Physics (CIOMP) plans to manufacture an 8 m aperture space optical system. Confronted with the great challenge of optically testing such a large system, we are eager to explore alternative test methods that afford simplicity and low cost.

Taking cost into consideration, test methods which use small devices to test large optics, have also been widely studied, such as stitching auto-collimating test^8^, scanning pentaprism test and stitching test based on the Shack-Hartmann wavefront sensor. The stitching auto-collimating test requires a proper ratio for the full-aperture to sub-aperture size; otherwise, one needs too many stitching processes. For the James-Webb space telescope (JWST) of ∅6.5 m, three ∅1.5 m auto-collimating flat mirrors hung on the top of an optical test tower were used to carry out a sub-aperture stitching test^9–11^. However, the ∅1.5 m flat mirrors were possibly deformed for the test of the JWST in different fields. The scanning pentaprism test is a highly accurate test suitable for large, flat and near-paraboloidal mirrors and optical systems^12,13^. Since the rotations of the pentaprism have almost no effect on deflection angle for a light beam in the scanning direction, the scanning pentaprism test can be used to measure radial slope errors in the mirror profile or wavefront with high precision^14^. However, the large vertical rotation stage loading the scanning pentaprism test system must be extremely robust and strong, which limits the application for testing large-aperture space optical systems. The stitching test based on Shack-Hartmann wavefront sensor has been subject of rapid development in recent years^15–17^. The main advantages of this test are high accuracy and high dynamics for testing mirrors. However, what we are proposing here is to test space optical systems.

The scanning Hartmann test (SHT) has been regarded as a low-cost and high-efficiency test method for large-aperture space optical systems. This method was proposed and verified in 2017^18^. It has already been used to obtain high precision results with a simplified implementation and for a smaller optics. There were many advantages to testing large optical systems using the SHT. First, this test involves the use of a small apparatus to test large optics, which significantly reduces the testing cost. Second, it can utilize the imaging sensor of optical system to gather measured data to test a finished space optical system without need for another sensor. In contrast, an interferometer cannot be used to test a finished space optical system with a mounted imaging sensor. Third, the SHT can be used to measure the local slope of wavefront errors (WFE) of the optical system and retrieve the WFE of an optical system rather than only calculating the MTF. However, the pointing errors of the translation stage during the scanning process can severely affect the test accuracy^18^.

We propose that a multi-beam array should be used to scan the full aperture of an optical system rather than a single collimated beam. The measurement information in the overlapping zones is used to determine the pointing errors between adjacent sampling positions of the multi-beam array. Then, the true WFE is acquired by stitching together sub-apertures. This multi-beam array stitching method can not only maintain the advantages of the SHT but also be used to make accurate corrections for the imperfections produced during the translation stage. In this paper, we present a general description of the proposed technique and the underlying principle. Simulation results are also presented to verify the feasibility of the proposed method. Furthermore, we develop a multi-beam array stitching test system (MASTS), use it to test an actual space optical system of ∅800 mm and report the test results and discussion. The multi-beam array stitching method is demonstrated to be valid for testing a large-aperture space optical system.

## Test principle

### Basic principle

#### Principle of the scanning Hartmann test (SHT)

**Figure 1:**
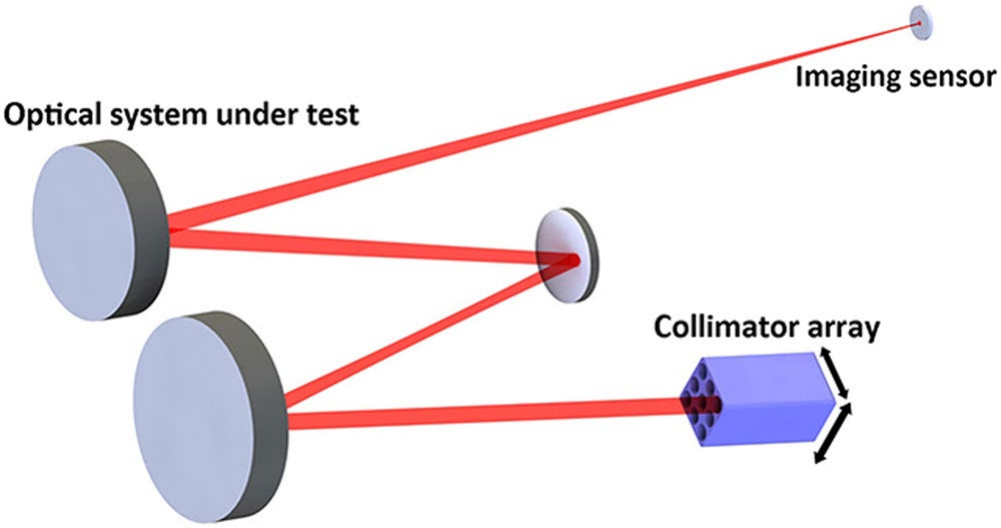
Sketch of the SHT.

The SHT for a large-aperture space optical system is based not on interferometry but on geometric properties. It provides a measurement of the WFE slopes of an optical system and uses them to retrieve the WFE. A sketch of the SHT is shown in Fig. 1, where the plane wave emitted by a collimator passes through the optical system and is focused onto a spot on the focal plane of the optical system. Depending on the position of the spot detected by the imaging sensor, the local slope (or tilt) of the WFE is determined. Subsequent analysis of all sub-aperture WFE leads to the determination of the overall WFE form^19^.

In the focal plane of the optical system, all the light spots generated by different sub-apertures are located at almost the same position. To avoid circumstance in which such spots are too close to be accurately resolved, measurements of each sub-aperture WFE should be conducted one by one. Thus, scanning with a collimator is used to measure all sub-aperture WFE. As the collimator mounted on the translation stage continuously scans the full aperture of the optical system along the given trajectory, all positions of the individual spot centroids are then recorded and compared with that of an ideal image point. Locational deviations between the spots and the ideal image point correspond to the WFE slope’s transverse aberrations of the homologous sub-apertures. Based on slope Zernike fitting, the WFE can be acquired by connecting measured WFE slopes of sub-apertures^20^.

#### Description of the multi-beam array stitching method

**Figure 2:**
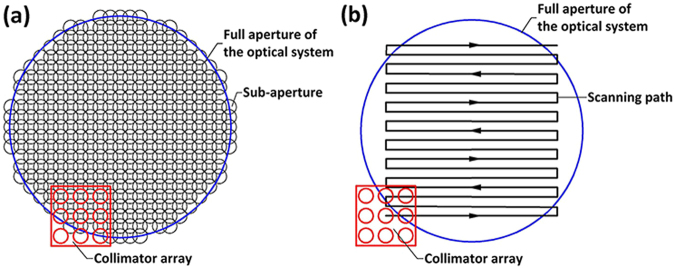
(**a**) Sub-aperture arrangement, (**b**) scanning path for the multi-beam array.

During the scanning process, the pointing errors due to the translation stage can lead to pointing variations for the collimated beam. The main obstacle for this scanning test mode is high sensitivity to pointing errors. The slopes for separately measured sub-apertures cannot be directly connected together due to the relative tilt errors. Hence, we propose that a multi-beam array should be used to scan the full aperture instead of a single collimated beam, as shown in Fig. 1. For one sampling position of the multi-beam array, several sub-aperture WFE can be successively measured, with the same pointing error. The sub-aperture arrangement and scanning path of the multi-beam array are shown in Fig. 2. By scanning with a multi-beam array, one sub-aperture can be scanned and aligned repeatedly by using different collimators. The sub-aperture WFE that has been measured should be measured again rather than skipped. Such repeat measurement information in the overlap zones determines the pointing errors between adjacent path points of the multi-beam array and is used to correct the residual pointing errors of the translation stage. Then, the reconstructed WFE of the optical system and the pointing errors between adjacent sampling positions of the multi-beam array can be simultaneously acquired through the stitching algorithm.

### Wavefront reconstruction and stitching algorithm

Low-order aberrations in the wavefront could be expressed by a linear combination of 37 Zernike polynomials^21^. Based on the measurement data of local wavefront slopes, the wavefront can be reconstructed through slope Zernike fitting. However, due to the pointing errors of the translation stage, the measured wavefront slopes are not at the same benchmark and show relative tilts. If the slope data were directly used to reconstruct the wavefront by slope Zernike fitting, the relative tilts would be coupled with the wavefront information, leading to the wrong reconstructed result. Therefore, we apply the stitching algorithm to decouple the relative tilts from the wavefront information. The stitching algorithm is based on slope Zernike fitting and a continuous slope condition. On the basis of 37 slope Zernike polynomials, the tilt terms corresponding to every sampling positions for the multi-beam array are introduced into the model fit. There are as many tilt terms as there are sampling positions for the multi-beam array. If the measured wavefront is smooth enough and shows continuous partial derivatives in the X and Y directions, the wavefront can be accurately described by 37 Zernike polynomials. Then, the relative tilt coupling in the measured data is almost described by the introduced tilt terms introduced and can be separated out from the measured data. Fortunately, only the low-order aberrations of the optical system can be measured for a lower sampling density and a larger sub-aperture aperture because the wavefront slope to be measured represents the average result of low-, middle-, and high- frequency wavefronts of the corresponding sub-aperture. The measured wavefront mainly represented the low frequency aberration after the mean filter. In other words, the measured slope data used in the stitching algorithm were continuous. Therefore, the reconstructed wavefront can be accurately described by 37 Zernike polynomials.

The wavefront reconstruction algorithm is based on the slope Zernike polynomials. The wavefront ${\rm{\Phi }}(x,y)$ can be expressed as

1 $${\rm{\Phi }}(x,y)=\sum _{k=1}^{n}{C}_{k}{Z}_{k}(x,y)+\varepsilon ,$$

where *C*_*k*_ are the coefficients of the Zernike polynomial, *Z*_*k*_(*x*, *y*) are Zernike polynomials, and *ε* is the residual of fitting.

The average slopes for the sub-aperture wavefront in the X and Y directions are expressed as

2 $$\{\begin{array}{c}{S}_{x}=\frac{1}{A}{\iint }_{A}\frac{\partial {\rm{\Phi }}(x,y)}{\partial x}dxdy;\\ {S}_{y}=\frac{1}{A}{\iint }_{A}\frac{\partial {\rm{\Phi }}(x,y)}{\partial y}dxdy,\end{array}$$

where A is the area of the sub-aperture.

Then, one can substitute formula (1) into formula (2). We can set up the relationship between the average slopes and the slope Zernike polynomial coefficients:

3 $$[\begin{array}{cccc}\frac{\partial {Z}_{11}(x,y)}{\partial x} & \frac{\partial {Z}_{12}(x,y)}{\partial x} & \cdots  & \frac{\partial {Z}_{1n}(x,y)}{\partial x}\\ \vdots  & \vdots  & \vdots  & \vdots \\ \frac{\partial {Z}_{N1}(x,y)}{\partial x} & \frac{\partial {Z}_{N2}(x,y)}{\partial x} & \cdots  & \frac{\partial {Z}_{Nn}(x,y)}{\partial x}\\ \frac{\partial {Z}_{11}(x,y)}{\partial y} & \frac{\partial {Z}_{12}(x,y)}{\partial y} & \cdots  & \frac{\partial {Z}_{1n}(x,y)}{\partial y}\\ \vdots  & \vdots  & \vdots  & \vdots \\ \frac{\partial {Z}_{N1}(x,y)}{\partial y} & \frac{\partial {Z}_{N2}(x,y)}{\partial y} & \cdots  & \frac{\partial {Z}_{Nn}(x,y)}{\partial y}\end{array}]\cdot [\begin{array}{c}{C}_{1}\\ {C}_{2}\\ \vdots \\ {C}_{n}\end{array}]=[\begin{array}{c}{S}_{x1}\\ \vdots \\ {S}_{xN}\\ {S}_{y1}\\ \vdots \\ {S}_{yN}\end{array}],$$

where n is the number of Zernike polynomials and *N* is sample number. Formula (3) is simplified as

4 $$ZC=S,$$

where *S*_*x*_ and *S*_*y*_ are the average slopes of the sub-aperture wavefront in the X and Y directions. However, the measured data include not only slope data of the wavefront but also pointing errors for the translation stage: ${M}_{x}={S}_{x}+{T}_{x}$ and ${M}_{y}={S}_{y}+{T}_{{\rm{y}}}$. After the tilt terms are introduced, formula (4) can be rewritten as

5 $$[Z,D][\begin{array}{c}C\\ {\rm{\Theta }}\end{array}]=[S+T],\,{\rm{with}}\,{D}_{2N,2m}=[\begin{array}{cccc}1 & 0 & \cdots  & 0\\ \vdots  & 0 & \cdots  & 0\\ 0 & 1 & \cdots  & 0\\ 0 & \vdots  & \cdots  & 0\\ 0 & 0 & \cdots  & 0\\ 0 & 0 & \cdots  & \vdots \\ 0 & 0 & \cdots  & 1\end{array}],{\rm{\Theta }}=[\begin{array}{c}{\theta }_{x1}\\ \vdots \\ {\theta }_{xm}\\ {\theta }_{y1}\\ \vdots \\ {\theta }_{ym}\end{array}],T=[\begin{array}{c}{T}_{x1}\\ \vdots \\ {T}_{xm}\\ {T}_{y1}\\ \vdots \\ {T}_{ym}\end{array}].$$

Here, m is the sampling number of the translation stage. The number of ‘1’s in each row of matrix D depends on the number of valid collimated beams at each sampling position.

We use the least squares method to solve the equations, namely,

6 $$[\begin{array}{c}C\\ {\rm{\Theta }}\end{array}]={({[Z,D]}^{T}[Z,D])}^{-1}{[Z,D]}^{T}(S+T),$$

where vector C corresponds to the coefficients of the Zernike polynomials for the reconstructed wavefront, and vector Θ corresponds to the coefficients of the terms. After vector C is brought into formula (1), the wavefront deformation Φ(*x*, *y*) can be calculated.

### Simulation analysis

To verify the principle feasibility of the test and the high precision of the stitching algorithm, simulations were conducted based on MATLAB® and ZEMAX®. MATLAB was used as a main program to offer instructions, and ZEMAX worked as a ray tracing arithmetic unit to provide feedback^22^.

**Figure 3:**
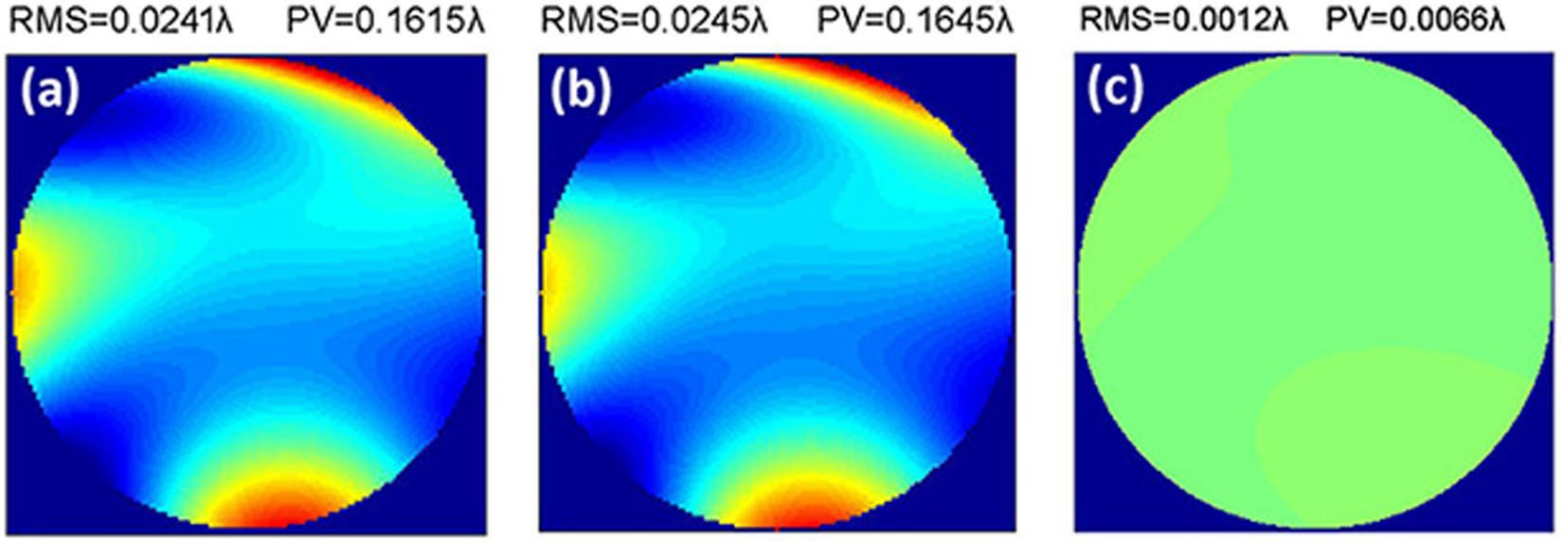
(**a**) Ideal WFE map of the optical system, (**b**) WFE map for the simulated test without including translation stage errors, (**c**) WFE map for the difference between the simulated WFE and the ideal WFE.

In ZEMAX, an off-axis three-mirror optical system was simulated with an entrance pupil diameter of 1400 mm, an exit pupil aperture of 186 mm, and a focal length in excess of 10 m. For a field of (0.3°, 0.7°), an ideal WFE map is shown in Fig. 3(a). The root mean square (RMS) of the WFE is 0.0241 λ (λ = 632.8 nm), and the peak to valley (PV) is 0.1615 λ. The test device in the simulation consisted of a collimated beam array with 3 rows and 3 columns, with the aperture for a single beam and the separation distance between beams set to 50 mm and 102 mm, respectively. We strictly simulated the practical test process for traversal of the collimated beam array across the full aperture of the optical system and successively measured the local slopes for the WFE. The sampling density was 25 × 25. On the one hand, we assumed that the translation stage was perfect and had no motion errors. The measured local slopes were used to fit the WFE based on the first 37 Zernike modes. The WFE was generated via a combination of the first 37 Zernike modes, as shown in Fig. 3(b). The simulated WFE map was basically identical to the ideal WFE map calculated using ZEMAX. The RMS of difference between the simulated WFE and ideal WFE was 0.0012 λ, as shown in Fig. 3(c).

**Figure 4:**
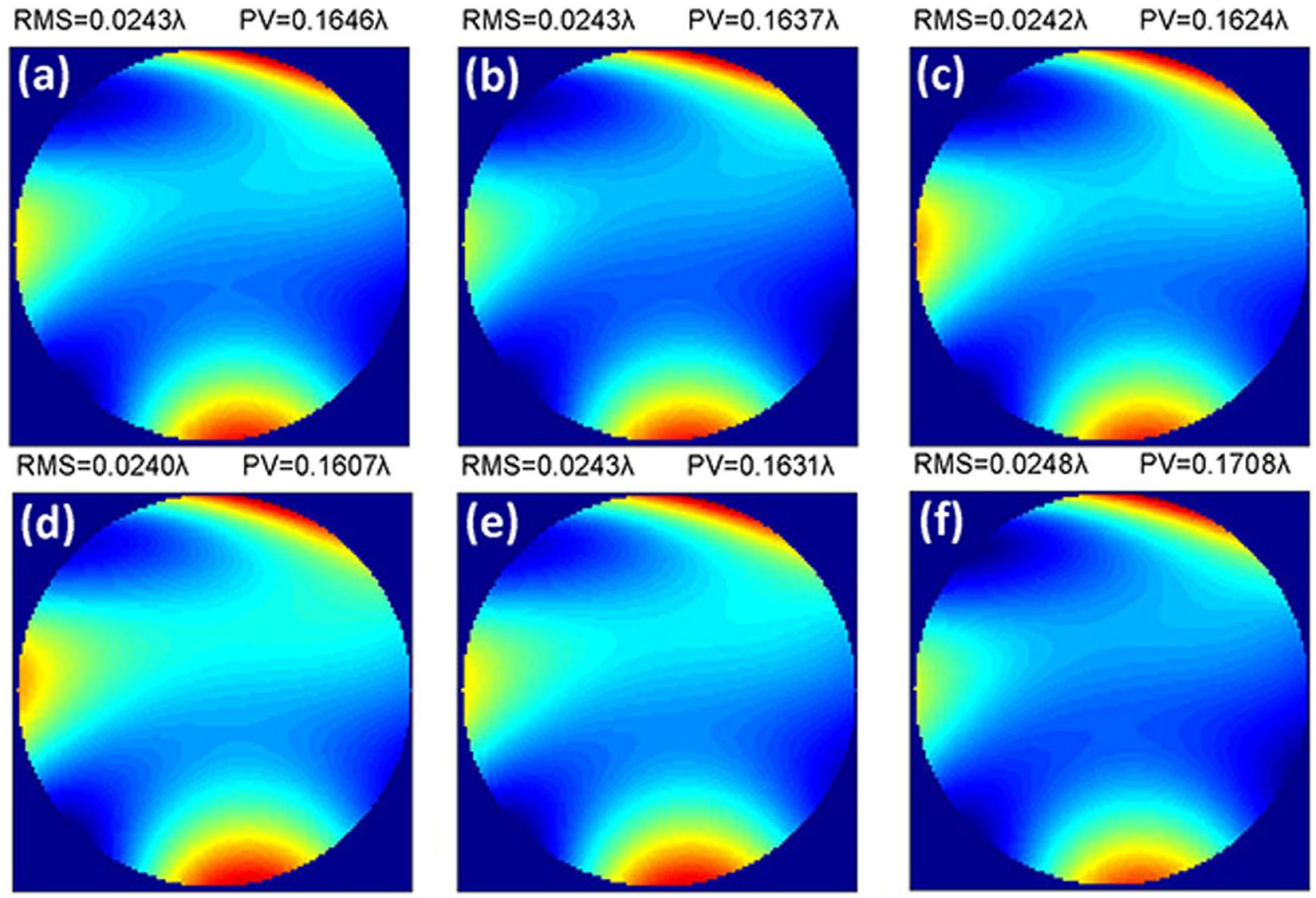
The WFE map for the optical system in fields of (0.3° + 1′, 0.7°), (0.3° − 1′, 0.7°), (0.3°, 0.7° + 1′), (0.3°, 0.7° − 1′), (0.3° + 1′, 0.7° + 1′) and (0.3° − 1′, 0.7° − 1′).

On the other hand, we took into consideration the pointing errors of the translation stage. The pointing errors will actually lead to two types of errors that include the pointing errors between adjacent sampling positions and the WFE in different fields. In practice, these two errors are coupled together and cannot be separated. However, to analyze the peculiar property of both, we respectively dealt with them in simulation. First, we checked the WFE map of the optical system for a field variation of approximately plus or minus one minute of arc (′). The results showed that WFE maps in fields of (0.3° + 1′, 0.7°), (0.3° − 1′, 0.7°), (0.3°, 0.7° + 1′), (0.3°, 0.7° − 1′), (0.3° + 1′, 0.7° + 1′) and (0.3° − 1′, 0.7°−1′) were generally the same, as shown in Fig. 4, which preliminarily indicated that the WFE was insensitive to a field error of no more than ± 1′. In a practical scanning process, the pointing error for the multi-beam array is certainly less than ± 1′. Therefore, we can ignore the effect of this field error.

**Figure 5:**
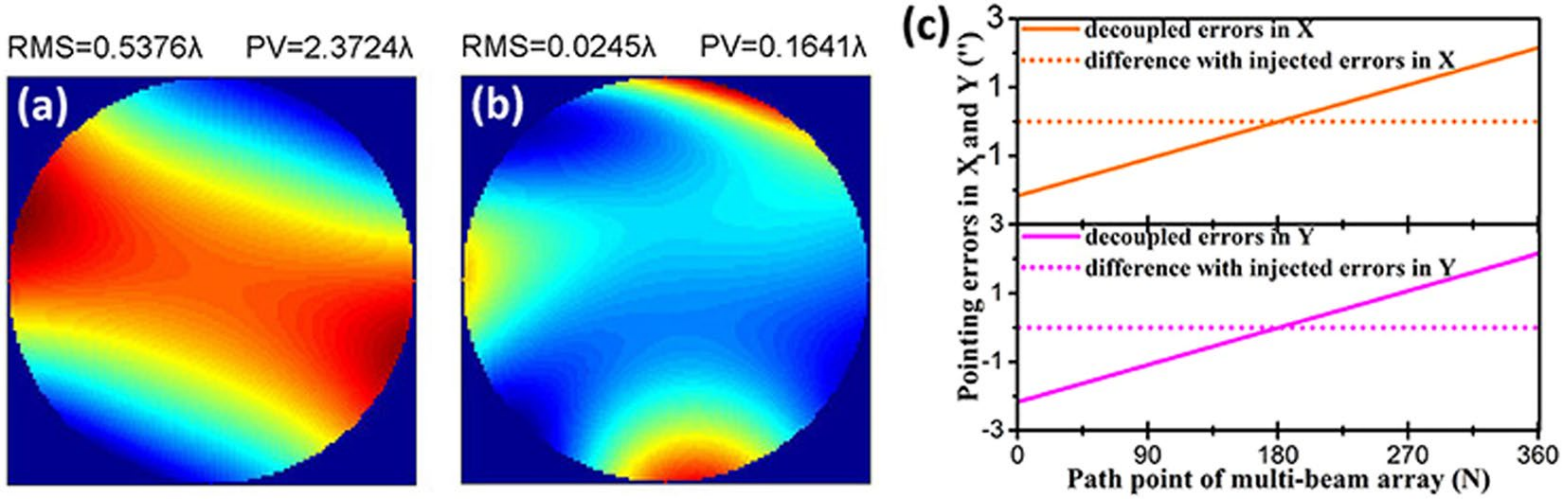
(**a**) WFE map for the simulated test with 0 to 4.3 seconds of arc (″) linear pointing errors, (**b**) WFE map for the simulated test with 0″ to 4.3″ linear pointing errors after correction by the stitching algorithm, (**c**) pointing errors decoupled from measured data.

Then, we analyzed the effect of pointing errors between adjacent sampling positions by simulation. We set 0 to 4.3 seconds of arc (″) linear pointing errors with the same field. The WFE map for the simulated test is shown in Fig. 5(a). Clearly, pointing errors of only 0″ to 4.3″ can cause significant variation in the WFE shape such that we cannot distinguish the low frequency aberration in the optical system from the test results^18^. Therefore, the use of the stitching algorithm to decouple pointing errors from the measured data was very significant in this test technology.

Finally, we added in the stitching algorithm to correct the imperfect wavefront shown in Fig. 5(a). The corrected WFE of the optical system and the pointing errors are acquired simultaneously, as shown in Fig. 5(b,c). The corrected WFE was restored to the ideal state and showed almost no difference in the RMS and PV compared with the WFE shown in Fig. 3(b), which demonstrated that the stitching algorithm has a sufficiently high precision to correct the residual pointing errors of the translation stage. The pointing errors in Fig. 5(c) grow linearly with a change in the path point following injection. The RMS of the difference between the decoupled pointing errors and the injected pointing errors was 2^–4^″.

The WFE reconstructions described above were all based on the first 37 Zernike modes. However, the limit in term of the number of modes to be reconstructed was not necessary 37. Instead, it depended on the aperture of the measured beam and spatial frequency sampled. The use of a sampling of 25 × 25 and a sub-aperture size of 50 mm enabled reconstruction of much higher modes. Then, simulations were conducted to check the reconstruction accuracy for higher modes.

**Figure 6:**
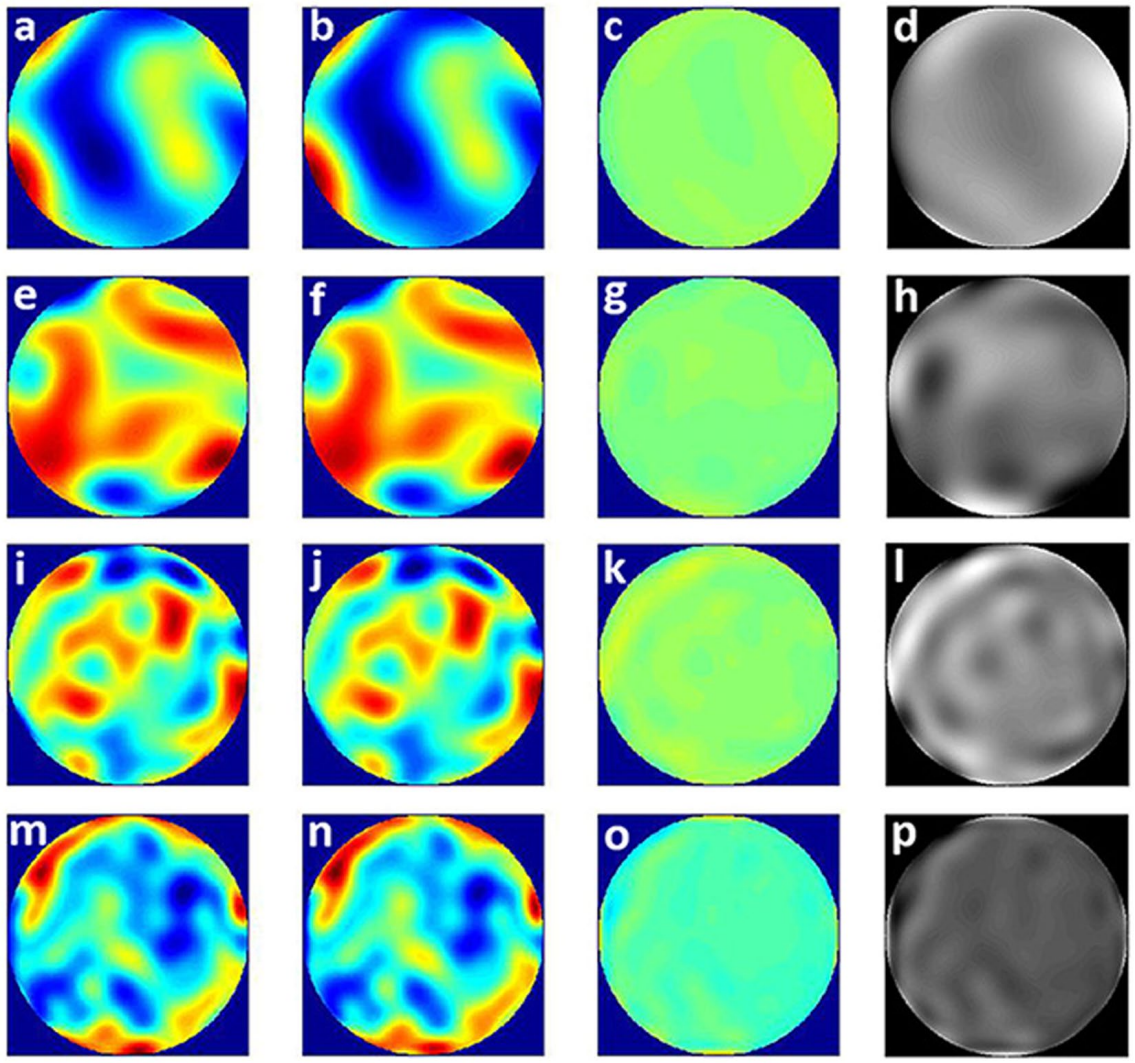
(**a**) Injected WFE map using 45 Zernike modes, (**b**) WFE map for the simulated test based on 45 Zernike modes, (**c**) difference between WFE (**a**,**b**). (**e**) Injected WFE map using 91 Zernike modes, (**f**) WFE map for the simulated test based on 91 Zernike modes, (**g**) difference between WFE (**e**,**f**). (**i**) Injected WFE map using 153 Zernike modes, (**j**) WFE map for the simulated test based on 153 Zernike modes, (**k**) difference between WFE (**I**,**j**). (**m**) Injected WFE map using 231 Zernike modes, (**n**) WFE map for the simulated test based on 231 Zernike modes, (**o**) difference between WFE (**m**,**n**). (**d**,**h**,**l**) and (**p**) represent the grey scale maps of WFE (**c**,**g**,**k**) and (**o**). The amplitude from white to black is equal to peak to valley.

Furthermore, we increased the Zernike items for the injected WFE to 231 to check the accuracy for the middle-frequency error. The injected WFE were generated respectively based on Zernike modes of 45, 91, 153 and 231. The injected WFE maps and simulation results were shown in Fig. 6.

Table 1 shows the simulation test results obtained using high-order Zernike modes. The reconstruction errors increased with the number of Zernike modes. When the number of Zernike modes used was more than 153, the difference between the input WFE and solved WFE was more than 11%. This verifies the reconstruction accuracy of much higher modes when sampling density is 25 × 25 and size of sub-aperture is 50 mm.

**Table 1:** WFE simulation results based on the use of high-order Zernike modes.

	Zernike 45	Zernike 91	Zernike 153	Zernike 231
Input WFE/RMS	0.3157 λ	0.3448 λ	0.2722 λ	0.4115 λ
Solved WFE/RMS	0.3214 λ	0.3513 λ	0.2763 λ	0.4143 λ
Δ/RMS	0.0219 λ	0.0254 λ	0.0300 λ	0.0609 λ
Δ/%	6.9%	7.4%	11.0%	14.8%

### Development of the multi-beam array stitching test system (MASTS)

The innovative metrology technology proposed here consists of four main components:

- The first one is the collimator array as an optical test device with high precision and stability.
- The second component is a high-precision four-axis translation stage for scanning and positioning. The collimator array is mounted on the translation stage to carry out 2D optical metrology.
- The third component is the plane array charge coupled device (CCD) with high resolution. The CCD is fixed onto the focal plane of the optical system to record the positions of the light spot emitted by the collimator array.
- The fourth component is a robust metrology software used for analyzing and treating the collected data, accurately reconstructing the WFE of the optical system under test, and correcting residual pointing errors of the translation stage.

**Figure 7:**
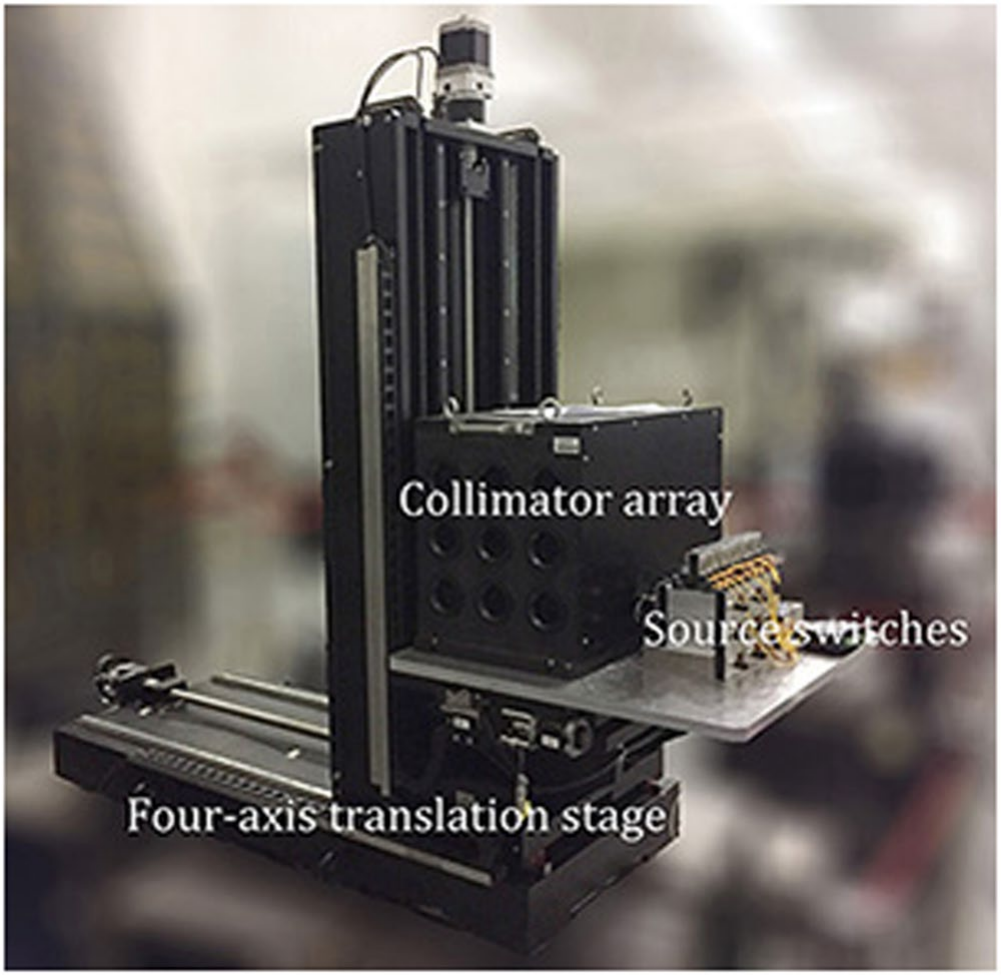
Four-axis translation stage loaded with a collimator array for carrying out bi-dimensional scans.

The collimator array was mounted onto the four-axis translation stage, as shown in Fig. 7. The four-axis translation stage can be used not only to position the platform but also to adjust the pitch angle and torsion angle of the collimator array. During the test, the computer automatically controlled the translation stage moving along the preset trajectory and residing at a path point. At each path point of the collimator array, the light sources for the effective beams (within the scope of the full aperture) were opened and closed in turn by switch controllers. To enable steady capture of the spot image, the light source was opened for one second before closing. The window method, threshold method and gray weighting method were adopted to accurately calculate the spot centroid^23,24^.

The parameters and performance of the main devices are described in Table 2.

**Table 2:** Parameters and performance of the main devices.

Collimator array with 3 rows and 3 columns	
Dimension	L = 610 mm × W = 336 mm × H = 420 mm
Aperture of a single collimator	∅50 mm
Distance between adjacent collimators	102 mm
WFE of a single collimator	better than 1/50 λ
Relative pointing errors of nine collimators	better than 0.3″
**four-axis translation stage**	
Maximum weight allowance	80 kg
Maximum movement distance in X and Z	900 mm
Guidance accuracy in X and Z	0.025 mm
Maximum angle of rotation in θ and η	±2.5°
Guidance accuracy in θ and η	2″
**Plane array CCD**	
Resolution	1624 × 1224
Sensor format	1/1.8″
Pixel size	4.4μm
Frame rate	15FPS

Since the diagonal dimension of the sensor was only 1/1.8″, the spots may move out of the range of the sensor with the continuous shift of the spots caused by pointing errors. Thus, we introduced a closed-loop error compensation system to ensure that the spot stayed within the detection range of the CCD. The position of the spot on the sensor was monitored in real time. We set the trigger boundary to be one-quarter of the sensor. When the spot moved onto the trigger boundary, it would automatically go back to the center via adjustment of the pitch and torsion angles of the collimator array. At the end of the scan and measurement, the aberration and WFE maps were automatically calculated and generated.

Theoretically, this method does not require a large number of sub-apertures. However, in practice, the random noise will be reduced with increasing number of sub-apertures. Therefore, a lower sampling density was always adopted first to increase efficiency. The number of sub-apertures can be increased depending to the actual effect of the random error.

The slope dynamic range of the MASTS was related to the exit pupil distance of the optical system under test and the size of the sensor. The relationship between these variables is described as

7 $$L\cdot \,\tan \,\theta =d/2,$$

where *L* is the exit pupil distance of the optical system, *θ* is the WFE slope, and *d* represents the size of the sensor. For example, if the exit pupil distance of the optical system to be tested is 2000 mm, the maximum slope θ is 2.083 mrad.

### Performance of the multi-beam array stitching test system

**Figure 8:**
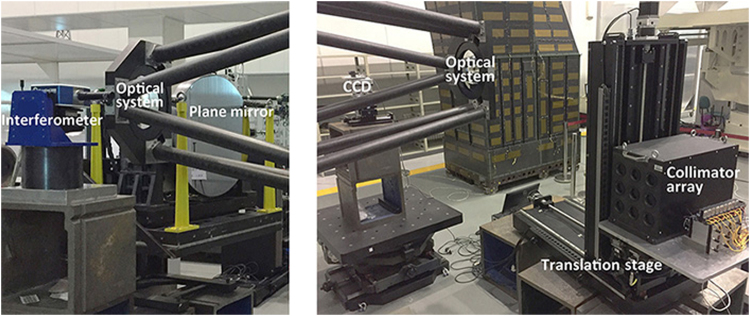
Layout of the auto-collimating test (left) and MASTS (right).

To demonstrate the capabilities of the MASTS, we measured the WFE of an off-axis three-mirror optical system and compared the results with those obtained by a recognized auto-collimating test^25^. The optical system to be tested was the same as the one described in the simulation analysis section. Since the diameter of the auto-collimating plane mirror was only 800 mm, we accordingly tested an 800 mm aperture with the MASTS. The layouts for the auto-collimating test and MASTS are shown in Fig. 8. The whole test was conducted on a large vibration isolation platform. The sampling density of the measured sub-aperture was 23 × 23. The scanning step size for the collimator array was 34 mm. The number of path points for the collimator array was 290(17 × 17). The total test time was approximately one hour.

**Figure 9:**
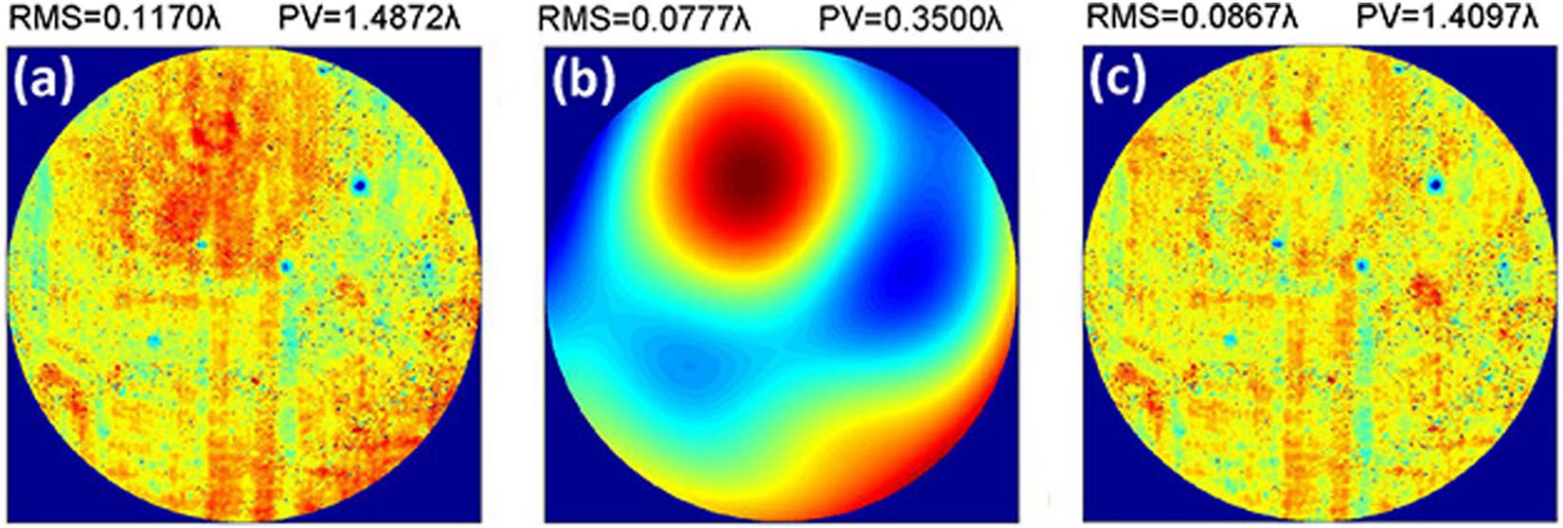
(**a**) WFE map measured by the auto-collimating test, consisting of (**b**) the first 37 polynomials Zernike fit and (**c**) the residual error.

**Figure 10:**
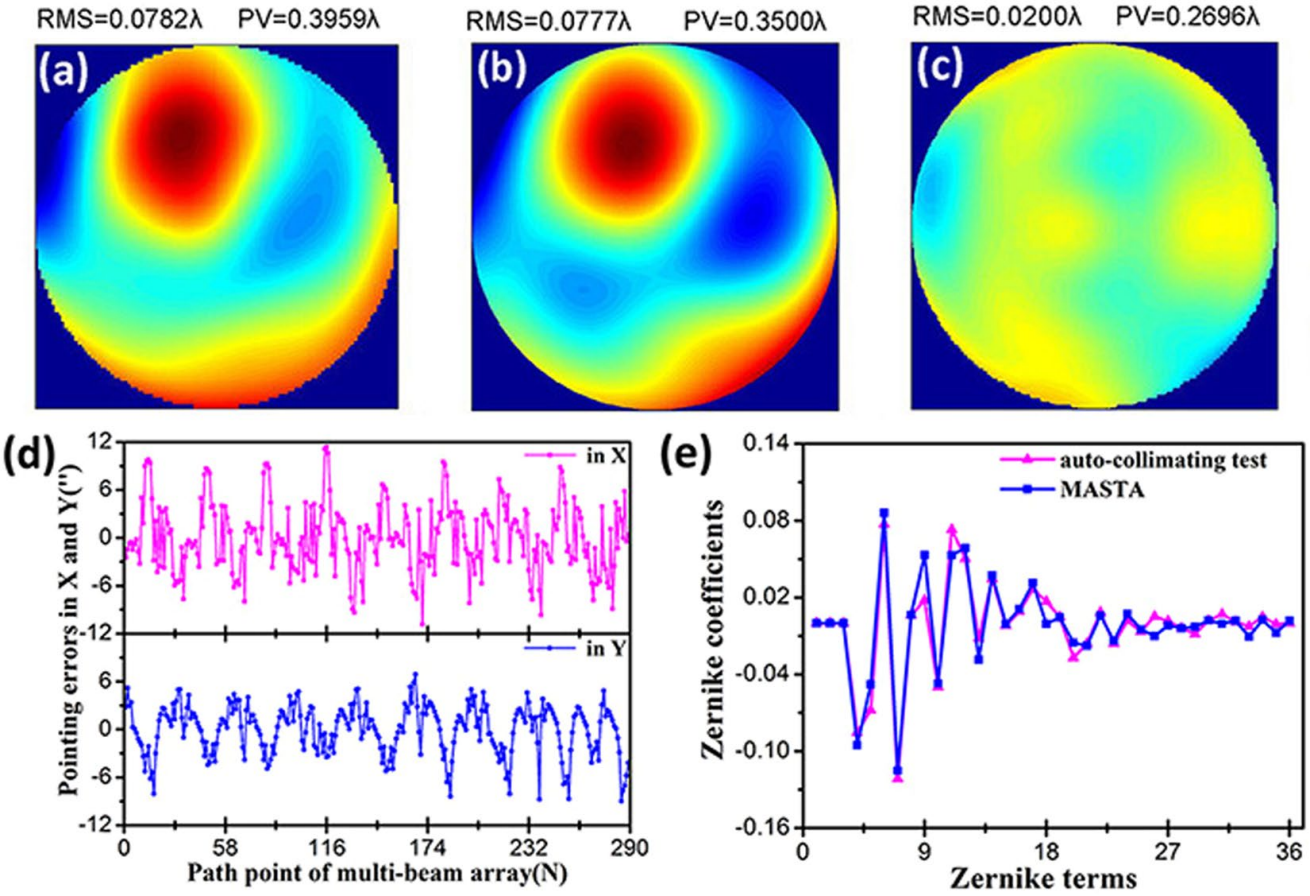
(**a**) WFE map measured with the MASTS, (**b**) WFE map of the first 37 Zernike modes measured by the auto-collimating test, (**c**) WFE difference between the auto-collimating test and the MASTS, (**d**) line and scatter graphs for pointing errors of the collimator array with changing path point, (**e**) coefficients of the Zernike terms for the auto-collimating test and the MASTS.

Figure 9 shows the WFE map measured by the recognized auto-collimating test. Figure 10(a) shows the WFE map measured with the MASTS. The fitting basis was the first 37 Zernike polynomials. Since the main aim of the optical system test stage was to test the aberration introduced by alignment errors and low-order deformations of the surfaces, reconstruction of the first 37 Zernike modes met the needs for image quality evaluation. Though the optical system showed middle-, high-frequency aberration, the data measured by the MASTS only contained information for the low-frequency aberration^26^, as analyzed in the stitching algorithm section. This can be used to further increase the stitching accuracy for low-frequency aberration.

After comparing the WFE map of the MASTS in Fig. 10(a) with that of the auto-collimating test shown in Fig. 10(b), we found excellent agreement between the two WFE maps. Then, we subtracted the WFE of the auto-collimating test from that of the MASTS. The difference in both is shown in Fig. 10(c). The RMS value of the WFE difference was better than 1/50 λ, demonstrating the high precision of the MASTS.

Figure 10(d) shows the decoupled pointing errors for the collimator array for the whole scanning process. Due to the regulation of the closed-loop error compensation system, the pointing errors were significantly reduced to ±12″. Moreover, the WFE caused by field variation in the range of ±12″ were very small and showed almost no effect on the test results.

The relationship between the pointing error of the measured beams and the effective focal length of the optical system was found to be similar to formula (7). It can be described as

8 $$f\cdot \,\tan \,\theta =d/2,$$

where *f* is the focal length of the optical system, *θ* is the pointing error, and *d* represents the size of the sensor. The results showed that the maximum pointing error of the translation stage was approximately ±12″. One can substitute the actual dimension of the sensor and the maximum pointing error into formula (8). The MASTS can be used to test optical systems with a focal length of less than 71600 mm, provided the CCD is not replaced.

As shown in Fig. 10(d), the two sets of coefficients of the Zernike terms for the auto-collimating test and the MASTS were very consistent, especially the fourth to eighth Zernike terms, which, respectively, represented vertical astigmatism, oblique astigmatism, horizontal coma, vertical coma, and primary spherical. For a space optical system test, we are mostly concerned with low-order aberration, such as primary spherical, primary astigmatism and primary coma. Therefore, the accurate test results for low-order aberration met our requirement for evaluating the imaging quality of optical systems and demonstrated the validity of the MASTS.

We conducted an additional five identical tests to verify the repeatability. The five tests were conducted on five different days under the same test conditions. We did not intentionally take other steps to maintain a thermostatic and isolated ambient. The five test results for RMS and PV are shown in Table 3.

**Table 3:** Repeat test results of RMS and PV for the MASTS.

No.	RMS (λ)	PV (λ)
1	0.0854	0.4295
2	0.0786	0.4059
3	0.0770	0.3946
4	0.0842	0.4386
5	0.0825	0.3985

The RMS deviation for the repeat test was expressed as follow:

9 $$D=\frac{1}{n}\sum _{k}RMS({W}_{k}-\overline{W}),k=1,2,\cdots ,n.$$

Then, the RMS deviation of the five tests was calculated to be 0.0120 λ, i.e., better than 1/80 λ, which demonstrated very high repeatability. In contrast, the recognized interferometric test required stringent ambient conditions to ensure steady test results because the long light path intensified the effect of airflow disturbance. Our interferometric test was conducted on a large vibration isolation platform in the dead of night. Even so, we should still continuously measure dozens of WFE maps and then choose maps showing the same characteristics and good repeatability as realistic results. Therefore, the MASTS shows a better capacity for resisting disturbance, which is advantageous, especially for the outdoor test of a space optical system.

## Conclusions

The multi-beam stitching method proposed in this paper is a valid test method for testing large-aperture space optical systems. Such a system not only maintains the advantages of the SHT but also enables accurate corrections to be made for imperfections produced during the translation stage by stitching together data obtained by successive sub-aperture acquisition. Simulation analysis showed that the stitching algorithm demonstrated high precision. Furthermore, the independently developed test system was successfully used to test an actual optical system of ∅800 mm. Compared with the results of the recognized auto-collimating test, the RMS for the WFE difference was better than 1/50 λ, demonstrating high accuracy for the MASTS. From repetitive tests, the RMS of the WFE deviation for five tests was better than 1/80 λ, which demonstrated high repeatability and low sensitivity to air turbulence compared to interferometric measurement. In conclusion, the MASTS shows great advantages of high accuracy, stability, and reliability for testing large-aperture space optical systems. The successful application of the proposed system for testing an optical system of ∅800 mm highlights great potential to solve test problems for space optical systems with ultra-large apertures in the future.
